# Using nominal group technique to identify and prioritize barriers to decentralizing HIV care to primary health centers in Lima, Peru

**DOI:** 10.1186/s12913-025-12618-8

**Published:** 2025-03-29

**Authors:** David Oliveros, Kelika A. Konda, Lynn M. Madden, Juan José Montenegro-Idrogo, Karla T. Tafur Quintanilla, Karin Sosa Barbarán, Benjamin M. Nikitin, Matthew Ponticiello, Carlos M. Benites, Jorge Sánchez, Frederick L. Altice

**Affiliations:** 1 Section of Infectious Diseases, Department of Internal Medicine, Yale School of Medicine, Yale University, New Haven, CT USA; 2 Center for Interdisciplinary Research on AIDS, Yale School of Medicine, Yale University, New Haven, CT USA; 3 Centro de Investigaciones Tecnológicas, Biomédicas y Medioambientales (CITBM), Universidad Nacional Mayor de San Marcos, Lima, Peru; 4 Department of Population and Public Health Sciences, University of Southern California, Los Angeles, CA USA; 5 APT Foundation, New Haven, CT USA; 6 Dirección de Prevención y Control de VIH-SIDA, Enfermedades de Transmisión Sexual y Hepatitis (DPVIH), Ministerio de Salud (MINSA), Lima, Peru

**Keywords:** Decentralization, HIV, Implementation science, Nominal group technique, RE-AIM framework

## Abstract

**Background:**

Decentralizing HIV services is an evidence-based strategy recommended by the World Health Organization to expand healthcare access by shifting most HIV care from specialty (SHCs) to primary health centers (PHCs) with the goal of maximizing health outcomes. To accelerate Peru Ministry of Health’s 2020 priority to transition from specialty to primary health centers, we assessed multilevel stakeholders’ perspectives on barriers and opportunities for scaling-up decentralization of HIV care.

**Methods:**

Between January and March 2024, we used nominal group technique (NGT), a mixed-methods research strategy, to rapidly identify barriers and rank potential solutions to decentralizing HIV services among two groups of patients (*N* = 16) and four groups of healthcare providers (*N* = 49) in Lima, Peru. Patient groups were those who: (1) were established in HIV care at a SHC; and (2) transferred from a SHC to a PHC. Three provider groups were mixed and included individuals from PHCs and SHCs while one was from a SHC in Central Lima. After listing all perceived barriers and solutions, participants in each group rank-ordered responses to generate potentially actionable responses.

**Results:**

Among 195 votes from 65 participants, multilevel HIV stigma was the highest priority barrier to decentralizing HIV care among both patients and providers (34.4%). While patients and providers prioritized different issues, all NGT groups highlighted a general lack of confidence in the expertise of PHCs (21.0%), system-level or transfer logistic challenges (19.0%), insufficient infrastructure and capacity of PHCs to provide HIV treatment (15.9%), and a lack of patient-level support (9.7%) as other major barriers to HIV decentralization.

**Conclusions:**

While the Peruvian Ministry of Health has prioritized HIV care decentralization, achieving this goal remains challenging. Identified barriers require a range of implementation strategies to achieve decentralization goals, such as process improvement strategies to address stigma and logistical barriers to transferring patients, while educational meetings, including tele-mentoring or expert feedback, may address a lack of confidence in provider expertise at PHCs. Deployment of hub-and-spoke treatment models could enhance communication between experts at SHCs and PHCs and ensure that patient continuity of care is achieved.

## Background

Decentralizing HIV services is an evidence-based strategy recommended by the World Health Organization to increase healthcare access by shifting the majority of care from specialty health centers (SHCs) to primary health centers (PHCs) [1]. Systematic reviews from low- and middle-income countries (LMICs) suggest that decentralizing HIV care can lead to improved retention in care (RIC) [2–4], decreased mortality [2, 5], improved antiretroviral therapy (ART) coverage [2–4, 6, 7], and comparable or higher levels of viral suppression [8]. Scaling up the decentralization of HIV care can also reduce the burden on limited specialty providers, improving treatment delivery efficiency by lowering health system costs for comparable outcomes and allowing specialists to focus on complex cases [8, 9]. For patients, some of the benefits of decentralized care may include reduced transportation time to health facilities, reduced waiting times, and more personalized care, including addressing noncommunicable diseases as patients age, thereby reducing program-level demand characteristics and facilitating engagement in HIV care [10].

In Peru, all HIV services are free, but have been concentrated in large, hospital-based specialty settings, often with 3,000–6,000 people with HIV (PWH), representing an extremely centralized HIV care system that places substantial time, transportation, and economic demands on patients to receive care. While Peru has made great strides in HIV diagnosis, linkage to care, and reduced time to ART initiation, RIC and viral suppression levels remain suboptimal, with the former representing the largest implementation gap (33%) [11]. Since 2023, Peru has also been providing pre-exposure prophylaxis (PrEP) for free at PHCs and hospitals that also provide ART. The low levels of RIC and viral suppression among PWH, however, hamper treatment as prevention efforts, as evidenced by the increasing incidence of HIV in Peru, which opposes the current global trend [12].

In 2020, in response to low RIC and other health system challenges posed by the COVID-19 pandemic, the Peruvian Ministry of Health updated its guidelines to recommend the decentralization of HIV treatment services from SHCs to PHCs that have “trained human resources, adequate infrastructure, equipment, supplies, care records, and adequate referral systems” [13]. These updated guidelines are enacted through local health directives, or *Direcciones de Redes Integradas de Salud* (DIRIS; Integrated Health Network Directives), which must navigate HIV service delivery within local PHCs located in specific districts.

Despite the goal of decentralizing HIV care, multi-factorial challenges remain, including perceived inadequate HIV expertise at PHCs, mixed political will, and stigma in an HIV epidemic concentrated in key affected populations, especially transgender women and men who have sex with men [14]. Importantly, there are no evidence-informed tools or guidelines on how to decentralize HIV care, necessitating the assessment of both providers’ and PWH’s perspectives to identify potential barriers and opportunities for implementation that could inform ongoing efforts to transfer patients from specialty settings to PHCs.

## Methods

### Context

As part of a larger implementation trial, we used the PRISM/RE-AIM implementation framework to assess the barriers and facilitators to decentralizing HIV care in Lima, Peru, an urban sprawl of 12 million people that covers an area of 1,032 square miles and has a limited public transportation infrastructure [15]. Greater metropolitan Lima is divided into four large regions, plus Callao, each with dozens of districts that are administratively overseen by a DIRIS. Each DIRIS oversees healthcare facilities that vary in terms of resources and capabilities, ranging from level 1 to level 4. Among these, level 3 (PHCs) and level 4 (hospital-based clinics) settings that meet a minimum threshold of resources, including a physician trained in HIV care and available laboratory testing and pharmacies, are designated as sites that can provide HIV services. Laboratory services must be able to test for CD4 and viral load and pharmacies must manage ART and medications for the prevention of opportunistic infections. Non-specialist providers from PHCs usually receive a two-week on-site clinical training, or *capacitación*, in HIV care at a SHC, but these trainings do not include licensing or qualification procedures.

### Study design

We conducted focus groups using nominal group technique (NGT), a mixed-methods research strategy [16], to rapidly identify barriers and potential solutions to decentralizing HIV services with a focus on the inner context from the PRISM/RE-AIM framework of patients (who would be the recipients of decentralization) and providers (who would deliver the services). NGT has advantages over traditional focus groups by ensuring equal inputs from participants, reducing the influence of power dynamics, and providing immediate and actionable responses to guide implementation [17–19]. This was especially important given the highly hierarchical nature of healthcare delivery in Peru, where specialty doctors have primarily been the sole prescribers and gatekeepers of HIV care.

NGT involves a specific question, followed by a silent generation process of written ideas, round-robin feedback from each participant in the group, and a recording of each idea on a flipchart. All individual ideas are discussed and clarified before being recorded with the ultimate goal of generating consensus of ideas until there is saturation of ideas and no member of the group has any additional thoughts to contribute. At the end of each session, participants are asked to vote on the ideas that they consider of highest importance or priority, leading to a rank-ordering of NGT results.

### Nominal group technique recruitment & procedures

Between January and March 2024, NGT sessions were conducted in-person and in Spanish with six different groups of stakeholders as part of an ongoing stepped-wedge implementation trial supporting the decentralization of HIV care. Two of these groups consisted of patients receiving HIV care in Central Lima, either at a large SHC or a PHC, who were recruited through convenience sampling. Patient participants were approached by their medical provider or a research assistant during their scheduled HIV care visits and given more information about the study. Focus groups were then scheduled according to participants’ preferences and availability. The SHC patients had been established in HIV care at the SHC for more than two years, while those at the PHC had been successfully transferred from the SHC 4 to 17 years prior.

Four additional NGTs were conducted with healthcare staff, including specialists, nurses, and peer navigators working in HIV care. One of these groups consisted of staff members from the SHC, while the other three were mixed groups of healthcare staff from SHCs and PHCs. Healthcare providers were recruited at a meeting that assembled staff from sites providing HIV care in three districts in Lima (DIRIS *Centro*, *Norte*, and *Sur)*, including administrative representatives from each DIRIS.

NGT focus groups were co-facilitated by experts trained in NGT and fluent in English and Spanish. The NGT question was “What gets in the way of transferring a patient with HIV from a SHC to a PHC?” Due to time constraints, only three of the six groups were able to respond to the following question: “What would need to change in order to successfully transfer a patient with HIV from a specialty hospital to the primary care level?” Patients were asked this question to offer potential solutions to improving the transition from SHCs to PHCs, as some patients did not feel comfortable transferring their care. After idea saturation, each participant was allocated three votes, which they could distribute among the different ideas however they deemed best to select those of highest priority (i.e. they could cast all of their votes for one idea if desired). An average of 10 participants, ranging from six to sixteen, were included in each 60–90-minute NGT session. Informed consent was obtained from all of the participants in the study and patients were compensated 35 PEN (~ 10 USD) for their time and participation.

### Data analysis

Findings from the NGTs were written in Spanish and then translated to English and back-translated for better understanding [20]. Rank-ordered data was analyzed between January and April of 2024. We used thematic analysis and applied the sociecological model to categorize participants’ responses into five major categories: stigma, lack of confidence in the expertise of PHCs, system-level or transfer logistic barriers, insufficient infrastructure and capacity of PHCs to provide HIV treatment, and lack of patient-level support. These barriers were then used to populate the socioecological model across the individual, interpersonal, organizational, community, and public policy levels [21].

## Results

**Fig. 1:**
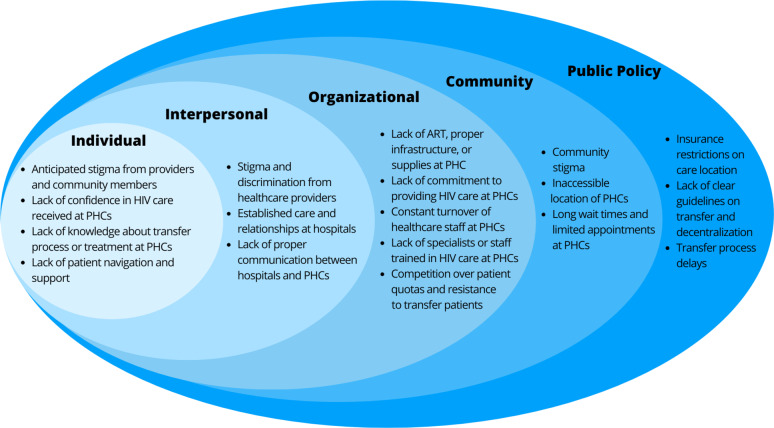
Socio-ecological model of barriers to HIV care decentralization

The results from NGT sessions on perceived barriers to decentralization of HIV services among patients are summarized in Table 1, while those among providers from SHCs and PHCs are summarized in Table 2. Figure 1 depicts selected barriers from each NGT session used to populate the sociecological model. The rank-ordering of NGT results on barriers to HIV decentralization is summarized in Table 3.

**Table 1:** Perceived barriers to decentralizing HIV services among people with HIV

“*What gets in the way of transferring a patient with HIV from a specialty health center to a primary health center?*”				
**#**	Patients established in HIV care at a Specialty Health Center (*n* = 9)	**Votes** ***N*** = 27 (%)*	Patients previously transferred from a Specialty Health Center to a Primary Health Center (*n* = 7)	**Votes** ***N*** = 21 (%)*
**1**	Community stigma and fear of being exposed as having HIV at PHC	11 (40.7)	Lack of confidentiality and community stigma at PHC	12 (57.1)
**2**	Established relationships and continuity of care at specialty hospital	4 (14.8)	Difficulties adapting to new health center and healthcare staff	3 (14.3)
**3**	No ART available at PHCs	3 (11.1)	Delays in transfer process and document processing	3 (14.3)
**4**	Lack of infectious disease specialist or trained staff at PHCs	2 (7.4)	Lack of training on management of HIV at PHC	2 (9.5)
**5**	Lack of proper infrastructure and laboratory supplies at PHCs	2 (7.4)	Difficulties obtaining appointments at PHC	1 (4.8)
**6**	Lack of peer navigators at PHCs	2 (7.4)	Distance or difficulty accessing PHC	0 (0)
**7**	Lack of privacy related to signaling or differentiation of HIV care at PHCs	1 (3.7)	Lack of proper infrastructure at PHC	0 (0)
**8**	Stigma and lack of discretion of staff at PHCs	1 (3.7)	Lack of specialists at PHC	0 (0)
**9**	Lack of confidence in the care received at PHCs	1 (3.7)	Lack of empathy from PHC staff with patient	0 (0)
**10**	Lack of specialty care for the treatment of comorbidities at PHCs	0 (0)		
**11**	Lack of empathy and training to treat key populations at PHCs	0 (0)		
**12**	Limited schedule and clinic hours at PHCs	0 (0)		

Legend: *PHCs* Primary Health Centers, *ART* Antiretroviral Therapy

*Each participant was allocated three votes, which they could distribute across ideas however they saw best

**Table 2:** Perceived barriers to decentralizing HIV services among specialty and primary health center providers

“*What gets in the way of transferring a patient with HIV from a specialty health center to a primary health center?*”								
**#**	Healthcare Staff at a SHC (*n* = 6)	**Votes** ***N*** = 18 (%)*	Mixed-Group of Healthcare Staff from SHCs and PHCs 1 (*n* = 12)	**Votes** ***N*** = 36(%)*	Mixed-Group of Healthcare Staff from SHCs and PHCs 2 (*n* = 16)	**Votes** ***N*** = 48(%)*	Mixed-Group of Healthcare Staff from SHCs and PHCs 3 (*n* = 15)	**Votes** ***N*** = 45(%)*
**1**	Stigma and discrimination from healthcare staff at PHCs	6 (33.3)	Concerns over lack of patient confidentiality at PHC and in home environment	8 (22.2)	Lack of proper infrastructure, laboratory equipment, and permanent human resources at PHCs	11 (22.9)	Perception from hospital doctors that complex cases will not be resolved at PHCs	8 (17.7)
**2**	Concerns over lack of confidentiality and fear of being exposed as having HIV in home environment	4 (22.2)	Not all PHCs have ART	7 (19.4)	Lack of confidence in the care received at PHCs	10 (20.8)	Concerns over lack of confidentiality and fear of being exposed as having HIV in home environment	8 (17.7)
**3**	Limited training of healthcare staff at PHCs	3 (16.7)	Patient feels like they receive better attention at the hospital	5 (13.9)	Lack of training on stigma, discrimination, and treatment of key populations at PHCs	9 (18.8)	Lack of proper communication between hospitals and PHCs	6 (13.3)
**4**	Lack of specialists and confidence in the care received at PHCs	2 (11.1)	Suboptimal referral and poor communication between SHCs and PHCs	4 (11.1)	Concerns over confidentiality and fear of being exposed as having HIV in home environment	6 (12.5)	Concerns over losing power by transferring patients	6 (13.3)
**5**	Lack of commitment to decentralization from leadership of PHCs	1 (5.5)	Resistance from doctors to transfer and from the patient to leave the health center	3 (8.3)	Lack of communication between hospitals and PHCs	5 (10.4)	Lack of proper infrastructure, laboratory equipment, and permanent human resources at PHCs	5 (11.1)
**6**	Lack of a proper patient flow and difficulty accessing services at PHCs	1 (5.5)	Lack of information and/or confidence in treatment received at PHCs	2 (5.5)	Lack of knowledge about transfer process or possibility of receiving ART at PHCs	4 (8.3)	Concerns over insurance coverage	4 (8.9)
**7**	Lack of specialty care for the treatment of comorbidities at PHCs	1 (5.5)	Long distances to receive care at PHCs	2 (5.5)	Delays related to the transfer process through the SIS	3 (6.3)	Constant turnover of healthcare staff at PHCs	2 (4.4)
**8**	Established relationships and continuity of care at specialty hospital	0 (0)	Insurance (SIS)-related restrictions on where the patient can receive care	2 (5.5)	Competition related to the need to fulfill patient quotas	0 (0)	Lack of confidence in the care received at PHCs	2 (4.4)
**9**	Constant turnover of healthcare staff at PHCs	0 (0)	Patient wants to remain where they are treated better	1 (2.8)	Difficulty accessing PHC from place of residence	0 (0)	Concerns over stigma from healthcare staff at PHCs	1 (2.2)
**10**	Delays related to the transfer process through the SIS	0 (0)	Long wait times and limited attention hours at PHCs	1 (2.8)	Movement of patients between places of residence complicates transfer and follow-up	0 (0)	Lack of training in HIV treatment for healthcare staff	1 (2.2)
**11**	Difficulty accessing PHC from place of residence	0 (0)	Lack of differentiated HIV service at PHCs	1 (2.8)	Established relationships and continuity of care at specialty hospital	0 (0)	Concerns over treatment of comorbidities and other health problems at PHCs	1 (2.2)
**12**	Better treatment and quality of care at SHC	0 (0)	Need to send patient to hospital for confirmatory test and treatment after diagnosis at PHC	0 (0)			Lack of patient knowledge about HIV treatment at PHCs	1 (2.2)
**13**	Lack of proper infrastructure and laboratory supplies at PHCs	0 (0)	Lack of a referral flow	0 (0)			Lack of knowledge about HIV treatment competencies at PHC	0 (0)
**14**	Differentiation of HIV services at PHCs	0 (0)	Fear, shame, and lack of confidence from patient to change care environment	0 (0)			Difficulty accessing PHC from place of residence	0 (0)

Legend: *SHCs* Specialty Health Centers, *PHCs* Primary Health Centers, *ART* Antiretroviral Therapy, *SIS* Integrated Health System (*Sistema Integrado de Salud*)

*Each participant was allocated three votes, which they could distribute across ideas however they saw best

**Table 3:** Rank-ordering of perceived barriers to decentralizing HIV care among patients and providers

Type of Barrier to HIV Care Decentralization	Votes					
	Patients (*n* = 16, 48 votes)	%	Providers (*n* = 49, 147 votes)	%	Total (*n* = 65, 195 votes)	%
Stigma	25	52.1	42	28.6	67	34.4
Lack of confidence in expertise at PHC	5	10.4	36	24.5	41	21.0
System-level or transfer logistics barriers	3	6.3	34	23.1	37	19.0
Insufficient infrastructure and capacity of PHCs to provide HIV treatment	5	10.4	26	17.7	31	15.9
Lack of patient-level support	10	20.8	9	6.1	19	9.7

Legend: *PHCs* Primary Health Centers

Patients and providers prioritized different sets of barriers. Among patients (*n* = 16), stigma was voted as the highest priority barrier (52.1%), followed by a lack of patient-level support (20.8%), a lack of confidence in the expertise at PHCs (10.4%), insufficient infrastructure and capacity of PHCs to provide HIV treatment (10.4%), and system-level or transfer logistic challenges (6.3%). Among providers from both SHCs and PHCs (*n* = 49), stigma was the highest priority barrier (28.6%), followed by a lack of confidence in the expertise at PHCs (24.5%), system-level or transfer logistic barriers (23.1%), insufficient infrastructure and capacity of PHCs to provide HIV treatment (17.7%), and a lack of patient-level support (6.1%).

The results from NGT sessions on potential solutions to barriers to decentralization are summarized in Table 4. Patients established in HIV care at a SHC prioritized the need to increase the availability of ART at PHCs (18.5%) as their primary solution, while those who were successfully transferred to a PHC prioritized the need to improve the confidentiality of ART services at PHCs (42.9%). Providers from the SHC prioritized the need to sensitize healthcare staff at PHCs (22.2%) and to improve the training for healthcare staff at PHCs to provide HIV treatment (22.2%).

**Table 4:** Proposed solutions to decentralizing HIV care

*“What would need to change in order to successfully transfer a patient with HIV from a specialty health center to a primary health center?”*						
**#**	Patients established in HIV care at a SHC (*n* = 9)	**Votes** ***N*** = 27(%)*	Patients transferred from a SHC to a PHC (*n* = 7)	Votes ***N*** = 21(%)*	Healthcare Staff at a SHC (*n* = 6)	Votes ***N*** = 18(%)*
**1**	Increase the availability of ART at PHCs	5 (18.5)	Different location and greater confidentiality of ART services	9 (42.9)	Sensitize healthcare staff at PHCs	4 (22.2)
**2**	Improve the infrastructure and privacy of PHCs	4 (14.8)	Digitalization of medical records to increase confidentiality and time management	4 (19.0)	Trainings for healthcare staff at PHCs to provide HIV treatment	4 (22.2)
**3**	Have infectious disease specialists at PHCs	4 (14.8)	Awareness campaigns that address HIV stigma	2 (9.5)	Improve the process of patient linkage, accompaniment, and peer navigation	2 (11.1)
**4**	Improve trainings for treatment of HIV for healthcare staff at PHCs	4 (14.8)	Reduce the bureaucracy of transfer process to decrease transfer time	2 (9.5)	Increase the involvement and commitment of the leadership of hospitals, PHCs, and DIRIS in the decentralization process	2 (11.1)
**5**	Implement more campaigns and information to destigmatize HIV in the community	3 (11.1)	Ongoing training on stigma and discrimination for ART staff at PHCs	2 (9.5)	Speed-up the administrative processes of referral through the SIS	2 (11.1)
**6**	Have different specialists to manage co-morbidities and other health problems at PHCs	3 (11.1)	Eliminate requirement for the renewal of transfer documentation	2 (9.5)	Create networks of support between general practitioners, specialists, and nursing staff	1 (5.6)
**7**	Improve quality of care and level of empathy of healthcare staff at PHCs	2 (7.4)			Implement adequate infrastructure needed to provide ART at PHCs	1 (5.6)
**8**	Improve counseling and peer navigation at PHCs	1 (3.7)			Greater commitment from staff at PHCs to provide quality care	1 (5.6)
**9**	Use reminders for appointments at PHCs	1 (3.7)			Increase the clarity of the transfer process for patients	1 (5.6)

Legend: *SHCs* Specialty Health Centers, *PHCs* Primary Health Centers, *ART* Antiretroviral Therapy, *SIS* Integrated Health System (*Sistema Integrado de Salud*), *DIRIS* Integrated Health Network Directives (*Direcciones de Redes Integradas de Salud*)

*Each participant was allocated three votes, which they could distribute across ideas however they saw best

## Discussion

To our knowledge, this is the first assessment and prioritization of barriers and facilitators to decentralizing HIV services in a LMIC early in the decentralization process, involving stakeholders like patients and providers. While previous studies on decentralization have mostly been conducted in rural settings such as Sub-Saharan Africa [22], pointing at comparable or higher RIC for PWH who started care at decentralized clinics [23], they seldom assess pre-implementation barriers.

In pre-implementation research, it is not surprising that distinct stakeholders either identify or prioritize barriers and solutions differently. When constructing alterative implementation strategies, it is important to incorporate those strategies that are key to better implementation. For example, in this study, the perceived barriers to decentralization differed substantially between patients and providers and even among patient groups, depending on where they receive HIV care. This discrepancy underscores the necessity of implementing locally-driven solutions to decentralization. We discuss how the identified barriers relate to the literature and potential opportunities to address them.

### Stigma as a major barrier to HIV care decentralization

Perceived stigma, in aggregate, ranked highest among all stakeholders as a barrier to decentralization, although it was higher for patients than for providers (52.1% vs. 34.4%). Stigma related to HIV is common, irrespective of setting [24], including in Peru [25], making it unsurprising that all groups identified this as a major concern. Although PWH in Peru have the autonomy to seek care at any facility that provides HIV services, PHCs tend to be smaller and closer to patients’ homes relative to SHCs, raising concerns about stigma and confidentiality.

Concerns about stigma were not limited to community members or other patients, but also included potential stigma and mistreatment by healthcare providers at PHCs. This is consistent with studies conducted in other contexts, which highlight a high prevalence of HIV-related stigma and discrimination by healthcare providers early in the decentralization process [26]. Nonetheless, other studies suggest that, as specialty services like HIV are integrated into primary care, increased interpersonal contact creates opportunities for stigma reduction among both patients and healthcare providers [27, 28].

At the organizational level, more focus is needed on stigma-reinforcing processes, such as differentiated or segregated HIV services, time and logistical demands on patients to receive medication, or protocols that otherwise label and devalue PWH [29]. One potential approach to reduce stigma, irrespective of PHC or SHC, is to consider behavioral design where choice architecture, nudging, and framing are used to reduce opportunities for stigma [30–33]. In the decentralization process, for example, comprehensive clinical transfer information may reduce discussions about risk behavior and reinforce a focus on successful viral suppression. Viral suppression could be framed to align with the very successful undetectable = untransmittable (U = U) educational campaigns [34]. Behavioral design interventions can also be integrated into blended implementation strategies like those from the Network for Improvement of Addiction Treatment (NIATx), which identify implementation gaps and opportunities for process improvement through a patient-centered approach of tailored organizational coaching [35–37].

### General lack of confidence in provider expertise at primary health centers

After stigma, participants prioritized concerns related to a perceived lack of confidence in the HIV expertise at PHCs (21.0%), which involved a perceived lack of training on HIV care, a lack of infectious disease or other specialists at PHCs, a perception that patients generally receive better care at SHCs, or that complex cases will not be competently addressed at PHCs. These concerns were substantially greater among providers (24.5%) than patients (10.4%), suggesting that patients may not have experienced a major difference in the care provided between the two sites. Physicians may have legitimate concerns, as PHCs in Peru are not typically designed to provide HIV or specialty care. Findings from mental health research suggest that PHCs generally have low confidence in treating specialty conditions like depression [38], a sentiment echoed by psychiatrists [39]. Absent from this conversation was recognition that PWH in Peru are growing older and, as incidence of noncommunicable diseases increases [40, 41], there needs to be an increase in the capacity to provide integral care for these conditions at the primary level.

In the face of educational or training deficits, it is crucial to select implementation strategies to overcome such barriers. The Peruvian Ministry of Health already requires physicians who care for PWH to receive training plus two-weeks of onsite supervision at a SHC. Participants from both SHCs and PHCs did not perceive this to be sufficient based on the ranking of this barrier, suggesting that supplemental support is needed. One potential solution is tele-education programs such as Project ECHO [42], which democratize specialty services through a virtual learning community that enhances knowledge and confidence in treating HIV with the goal of maintaining viral suppression [43, 44]. Alternatively, to address the perceived lack of experts in infectious diseases, many settings have revised clinical roles through task-shifting, especially in LMICs like Peru where there are considerable human resource limitations [40, 45]. Task-shifting allows nurses or other non-physicians to manage most HIV service delivery, and studies have found that such strategy does not result in suboptimal health outcomes [4, 46–48].

### System-level and transfer logistic barriers

System-level and transfer logistic barriers included a perceived lack of adequate communication between SHCs and PHCs, delays related to the transfer process through the Peruvian national health insurance (i.e., SIS - *Sistema Integrado de Salud*), insurance restrictions on care location in relation to some aspects of care, no documented guidelines on how to decentralize HIV care, resistance from SHC providers to transfer patients, reliance on SHCs for some kinds of testing, competition related to the need to fulfill patient quotas, and a lack of commitment from the leadership of PHCs to provide HIV care. While increasing political will from healthcare leadership might pose challenges, system-level interventions that seek to increase the clarity and efficiency of decentralization will facilitate the transfer of PWH, which could be accomplished by developing guidelines for decentralization [49].

Patients who had been successfully transferred to a PHC recognized the need to limit the bureaucratic complexity of the transfer process to reduce transfer time, as well as to eliminate requirements for the renewal of transfer documentation. Flowcharting and process improvement techniques may help overcome such barriers [50]. Healthcare providers also pointed to a need to speed-up administrative processes of referrals by increasing communication between SHCs and PHCs. From an implementation perspective, this barrier might be overcome by strengthening hub-and-spoke models that create a bidirectional network for communication and referral between SHCs and PHCs. These models have been successfully implemented in other contexts and might optimize the decentralization process [51, 52].

In addition to new implementation strategies, tools may be helpful for scaling-up decentralization. Such tools might include a patient transfer form that includes relevant clinical information, patient checklists to help select which patients are eligible for transfer, patient navigators to guide patients through the process, or electronic medical records that can be viewed across all sites. Currently, most PHCs still use paper medical records [53], which poses challenges for referral and coordination between primary and specialty care [40, 54, 55]. Although the Ministry of Health recently created the *Sistema de Información de Historia Clínica Electrónica* (SIHCE) to allow HIV clinicians to view basic information, this system lacks clinical narratives, summary data, and checklists and requires clinicians to input information in addition to maintaining paper records to ensure completeness.

### Insufficient infrastructure and capacity of primary health centers to provide HIV treatment

All groups highlighted concerns about insufficient infrastructure and capacity available within PHCs to provide adequate HIV treatment (15.9% overall). Elements of this concern included constant turnover of PHC staff, ART not being available onsite, lack of space dedicated to HIV care, or lack of laboratory supplies or testing at PHCs. This concern, however, was greater among providers (17.7%) than among patients (10.4%), similar to findings regarding HIV expertise at PHCs. Notably, patients who had been successfully transferred from a SHC to a PHC did not allocate any votes to this barrier.

Patients without experience at PHCs and clinicians generally ranked this barrier more highly, but this was in part due to misperceptions about PHCs, including their perception that ART was not available onsite (one of the requirements by the Ministry of Health to be an HIV clinic). This discrepancy between the policy and the knowledge of patients and providers represents a major barrier to HIV care decentralization, which might be better addressed through enhanced communication between SHCs and PHCs through a spoke-and-hub model or through case presentations during tele-education sessions.

### Lack of patient-level support

Patients were especially concerned about a lack of patient-level support available within PHCs (20.8%), which differed substantially for established and transferred patients. For example, transferred patients had the lived experience of challenges adjusting to how the new (i.e., PHC) clinic operated, whereas established but non-transferred patients generally appreciated the case management and other services available in much larger SHCs. While providers prioritized concerns over a lack of clinical expertise at PHCs, they did not view a lack of patient-level support as a major barrier to decentralization (6.1%), as they are not the recipients of HIV care and may be unfamiliar with the experiences of patients. These differences between patients and providers point to the need to incorporate patient perspectives in the delivery of HIV care and the decentralization process. One of the key principles in effective implementation involves understanding and addressing the perspectives of the customer, which in this case, involves the patient.

Patient concerns about continuing care at a new facility involved difficulties obtaining appointments at PHCs, a lack of knowledge about the transfer process, long wait times and limited hours at PHCs, and not knowing how to navigate the system. Peer navigation or orientation sessions about clinic operations may support decentralization and care engagement [56, 57], as can establishing a hotline or social networking app where operational issues are shared collectively [58].

### Limitations

Despite the significance of our findings, this study has limitations. We did not collect demographic information about NGT participants, so we do not know the distribution of variables such as age, gender, sexual orientation, or clinical role within each health facility. While NGTs provide a rich opportunity to learn extensively about the perspective of stakeholders, they only provide insights into what stakeholders believe and not necessarily what is true (e.g., ART availability). Additionally, where probing is not extensive, it may not extensively expand our understandings of stigma experiences and other barriers to decentralization. Finally, as has been shown elsewhere [59–61], barriers are likely to differ based on where stakeholders receive or provide care. For example, providers consisted of participants from three administrative districts of Lima that are at different stages of decentralization. It is therefore possible that separating participants by district would have elicited different results due to different sets of priorities. A more nuanced NGT by site might inform site-level barriers and implementation solutions may differ.

## Conclusions

Despite goals to decentralize HIV services in Lima, stakeholders within the inner context of PRISM highlight the need to address major barriers of multilevel HIV stigma, a general lack of confidence in the expertise of PHCs, system-level barriers such as administrative delays, insufficient infrastructure and capacity of PHCs to provide HIV treatment, and a lack of patient-level support. Addressing these barriers will require tailored organizational coaching that identifies implementation gaps and stigma-reinforcing processes along the HIV care continuum. Moreover, addressing a lack of communication and collaboration between the SHCs and PHCs will require strengthening hub-and-spoke networks of referrals and collaboration, potentially through tele-education models such as Project ECHO, which provide ongoing training for PHC staff to increase the confidence in managing HIV at PHCs.

## Data Availability

All data generated or analyzed during this study are included in this published article.

## Abbreviations

SHCs: Specialty health centers
PHCs: Primary health centers
ART: Antiretroviral therapy
RIC: Retention in care
LMICs: Low- and middle-income countries
PWH: People with HIV
DIRIS: Integrated Health Network Directives
NGT: Nominal Group Technique
SIS: Integrated Health System
SIHCE: Sistema de Información de Historia Clínica Electrónica
